# Quantifying the Human Impacts on Papua New Guinea Reef Fish Communities across Space and Time

**DOI:** 10.1371/journal.pone.0140682

**Published:** 2015-10-14

**Authors:** Joshua A. Drew, Kathryn L. Amatangelo, Ruth A. Hufbauer

**Affiliations:** 1 Department of Ecology, Evolution and Environmental Biology, Columbia University, New York, New York, United States of America; 2 Division of Vertebrate Zoology, American Museum of Natural History, New York, New York, United States of America; 3 Department of Environmental Science and Biology, The College at Brockport, State University of New York, Brockport, New York, United States of America; 4 Department of Bioagricultural Sciences and Pest Management, and Graduate Degree Program in Ecology, Colorado State University, Fort Collins, Colorado, United States of America; Department of Agriculture and Water Resources, AUSTRALIA

## Abstract

Describing the drivers of species loss and of community change are important goals in both conservation and ecology. However, it is difficult to determine whether exploited species decline due to direct effects of harvesting or due to other environmental perturbations brought about by proximity to human populations. Here we quantify differences in species richness of coral reef fish communities along a human population gradient in Papua New Guinea to understand the relative impacts of fishing and environmental perturbation. Using data from published species lists we categorize the reef fishes as either fished or non-fished based on their body size and reports from the published literature. Species diversity for both fished and non-fished groups decreases as the size of the local human population increases, and this relationship is stronger in species that are fished. Additionally, comparison of modern and museum collections show that modern reef communities have proportionally fewer fished species relative to 19^th^ century ones. Together these findings show that the reef fish communities of Papua New Guinea experience multiple anthropogenic stressors and that even at low human population levels targeted species experience population declines across both time and space.

## Introduction

Understanding the relative contribution of factors influencing the distribution of biodiversity is one of the major goals of ecology, one that takes on special significance when viewed through the lens of the 6^th^ mass extinction [1]. When areas of high biodiversity are found in close proximity to large human populations they face a suite of pressures occurring across multiple spatial scales. When that biodiversity is the focus of extractive exploitation, it becomes difficult to determine if the impacts on population sizes are direct (via activities such as hunting, fishing, timber harvesting) or indirect (via habitat loss, fragmentation and degradation). Determining the sources of population change in directly targeted species can be further complicated when those populations still bear the legacy of historical exploitation [2]. With limited conservation resources there are real opportunity costs incurred by management agencies when there is a mismatch between conservation action and population stressors. Thus, identifying specific population stressors is an important first step in crafting management plans.

The coral reefs of Papua New Guinea lie within the epicenter of marine biodiversity called the Coral Triangle [3,4]. The country is home to over 1500 species of coral reef fishes and at least 514 species of corals [5]. These reefs face threats at multiple scales, ranging from global increases in oceanic temperature [6] and ocean acidification, to local factors such as point and non-point source pollution, increased disturbance, and fishing pressure [7]. Many of these local threats are proportional to human population size [8,9]. Local conservation action must target these threats effectively, and a crucial first step to doing so is distinguishing the relative importance of general habitat degradation and fishing pressure.

**Fig 1:**
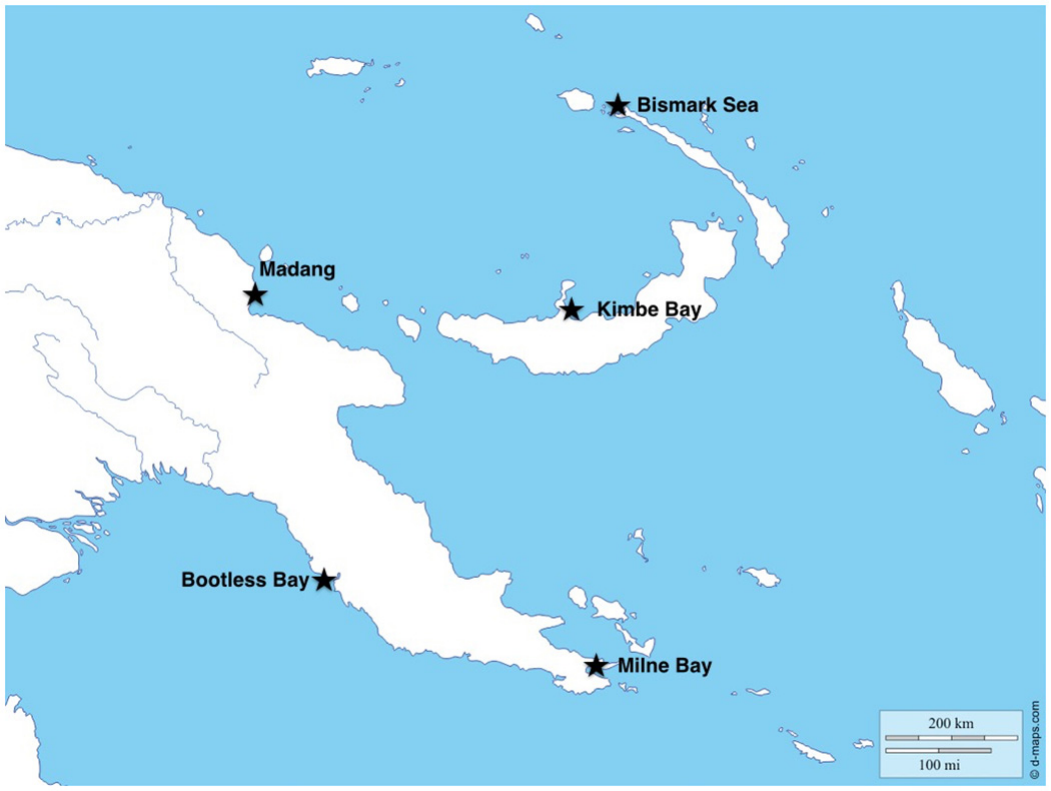
Location of sampling areas. Map downloaded and used with permission from http://www.d-maps.com.

Coral reefs in Papua New Guinea exist along a gradient of human population sizes from the heavily populated Bootless Bay to the remote Bismark archipelago (Fig 1). To better understand the influences on fish diversity in Papua New Guinea of both fishing *per se*, and the generalized effects of development and human population pressure, we can evaluate species diversity across this gradient of population pressures, distinguishing between species that are under pressure from fisheries and those that are not. Here, we compare the fish fauna of Papua New Guinea across five different localities covering both mainland Papua New Guinea (Madang, Milne Bay, Bootless Bay) and the Island of New Ireland (Kimbe Bay, Bismark Sea). We use the size of the human population as a proxy for both environmental degradation and fishery pressure, given that the majority of fisheries within this region are local (<50km) [10,11]. To test between direct exploitation from fisheries and the general effects of environmental degradation, we compare diversity among datasets of species defined as either fished or non-fished. To place the current community assembly of Bootless Bay in a historical context, we compare modern diversity to a historical baseline by incorporating data from museum collections made in Bootless Bay from 1881–1889. Lastly, we specifically focus on the relationships between human population size and species diversity for two groups of fishes that are particularly susceptible to fisheries pressure (groupers and sharks) across this human population gradient.

## Materials and Methods

Current species occurrences were taken from five published literature surveys [12,13,14,15,16] and recent species descriptions [17]. Historical museum data were obtained from the National Museum of Victoria, (www.fishnet2.net, accessed Feb 17, 2015).

The modern surveys were conducted using similar, but not identical survey techniques, and thus results should be interpreted with reasonable caution. To minimize potentially confounding effects of sampling effort across surveys and differences in survey methods, we excluded cryptic, nocturnal, interstitial, and/or easily confused species from the analyses, focusing on obvious, easily identifiable and more common species. Based on published surveys of fish markets [10,13] and our own observations in Papua New Guinea markets, we divided families into fished and non-fished datasets. We identified nine families that are commonly fished: Surgeonfish (Acanthuridae), Triggerfish (Balistidae), Fusiliers (Caesoindae), Jacks (Carangidae), Requiem sharks (Carcharinidae), Emperors (Lethrinidae), Snappers (Lutjanidae), Parrotfish (scarine members of Labridae), and Groupers (epinepheline members of Serranidae). We identified three families that are rarely fished: Butterflyfish (Chaetodontidae), Hawkfish (Cirrhitidae) and Damselfish (Pomacentridae). In addition we supplemented our fished dataset with species that were specifically mentioned as being common in Papua New Guinean fisheries [11]. All species names and taxonomic validity were evaluated using the Eschmeyer catalogue (http://researcharchive.calacademy.org/research/ichthyology/catalog/fishcatmain.asp). The resulting list contained 470 species in 29 families and 120 genera. Data are freely available from the Dryad Digital Repository (http://dx.doi.org/10.5061/dryad.41mk8)

Our filtering of the data does, by definition, reduce the taxonomic diversity relative to the raw data, and thus we acknowledge that our results reflect subsets of total reef fish diversity. This approach however does allow us to incorporate data from a variety of public data sets and to focus on species of conservation concern (but see caution below regarding comparing data generated using different methods).

To examine potential effects of the size of the human population at the five sites on species richness of the reef fish communities, we performed regressions analyses evaluating the ability of human population size to predict total species number, as well as numbers of fished and non-fished species. Raw population data were taken from the 2011 Papua New Guinea census; the most recently published data available (data available at https://spmt.files.wordpress.com/2010/09/preliminary-figures-census-2011-1.pdf). Because the range in human population size was large, population size data were log-transformed before analysis to improve normality of the residuals. As Bootless Bay has both the largest population size and the lowest species richness (Table 1) its influence on the regression is substantial. Thus, we also evaluated whether the pattern was consistent without including that site.

**Table 1:** Summary information for Papua New Guinea Fishes. Fished and Non-fished refer to the number of species within each of these categories. Fished species are >30 cm SL while non-fished are <30 cm SL.

Sample Location	Total Number of species	Fished	% Fished	Non Fished	% Non- Fished	Human Population (2011)
Bootless	167	95	56.89	72	43.11	364,145
Bismark Sea	330	205	62.12	125	37.88	23,000
Kimbe	339	213	62.83	126	37.17	16,300
Madang	349	212	60.74	137	39.26	42,000
Milne	393	242	61.58	151	38.42	12,628

To evaluate beta diversity among sites we calculated Jaccard distances between pairs of sites for each of the fished and non-fished datasets. Given that Bootless Bay has substantially lower species richness, which may drive beta diversity patterns, we decomposed beta diversity into its components of species richness and turnover/replacement [18,19]. We calculated total beta diversity and its components in R 3.1.1 using the BAT package [20]. We evaluated statistical differences between fished and non-fished datasets using the Wilcoxin Signed Test.

To evaluate how fish communities found at lower diversity sites compare to those found at higher diversity sites we performed nestedness analyses [21]. Nestedness analysis determines if lower diversity sites contain non-random subsets of species from higher diversity sites. In a perfectly nested set of sites (significant nestedness), all species found at the most species-rich site would be found at all other sites, and only the most frequently encountered species would be found at the most species-poor site. These analyses can also demonstrate significant anti-nestedness, where species at depauperate sites are non-random sets of species not necessarily found at more species rich sites. Anti-nestedness can result from a variety of different patterns of incidence, including non-inclusive sets, checkerboards (a measure of randomness in distribution), or gradients with high turnover [22]. We used the NODF nestedness metric for presence-absence data implemented in the FORTRAN95 software NODF-Program [23]. The NODF metric (Nested metric based on overlap and decreasing fill) calculated overall nestedness and the contribution of sites (columns) and species (rows) to the nestedness patterns. We used a ‘fixed-fixed’ null model for randomization of species presences that preserves row and column totals; these algorithms have low Type 1 error rates and good statistical power [21,24]. We selected a ‘proportional’ null model of full rows and columns that swapped rows and columns proportional to their constraints. We created 1000 random matrices for each run, and created those random matrices by performing swaps equal to 10 times the matrix sizes. We performed analyses for three different sets of fish species: all species, the fished dataset, and the non-fished dataset across the five sites. We also performed the analyses without Bootless Bay to evaluate the impact of that site on our results.

To evaluate the potential impact of fishing on species composition over time in Bootless Bay we compared data from historical and modern data sets. Specifically, we calculated the ratio of fished to non-fished species for Bootless Bay (present in both historic and modern data) and extirpated (present in historic but *not* in the modern dataset) fish species, as well as for the entire dataset. We then used a Chi-square test to evaluate the significance of the differences in the ratios.

Lastly, to examine the impact of human population pressure on species that are particularly affected by fisheries including (e.g. fisheries indicator species), groupers (epinepheline serranids) and sharks, we calculated the percent of the total number of species in each of these groups present at each site compared to the total number of species in each group found in the entire dataset.

## Results

We generated a list of modern species containing 470 species that we divided into two datasets, a fished dataset containing 302 species and a non-fished dataset containing 168 species (for full list see S1 Table). These species were distributed over five different localities of which Milne Bay had the highest species richness (N = 393) and Bootless Bay had the lowest (N = 167). There was a wide range of human population size among the sample localities with Milne Bay having approximately 5% the human population of Bootless Bay (Table 1).

**Fig 2:**
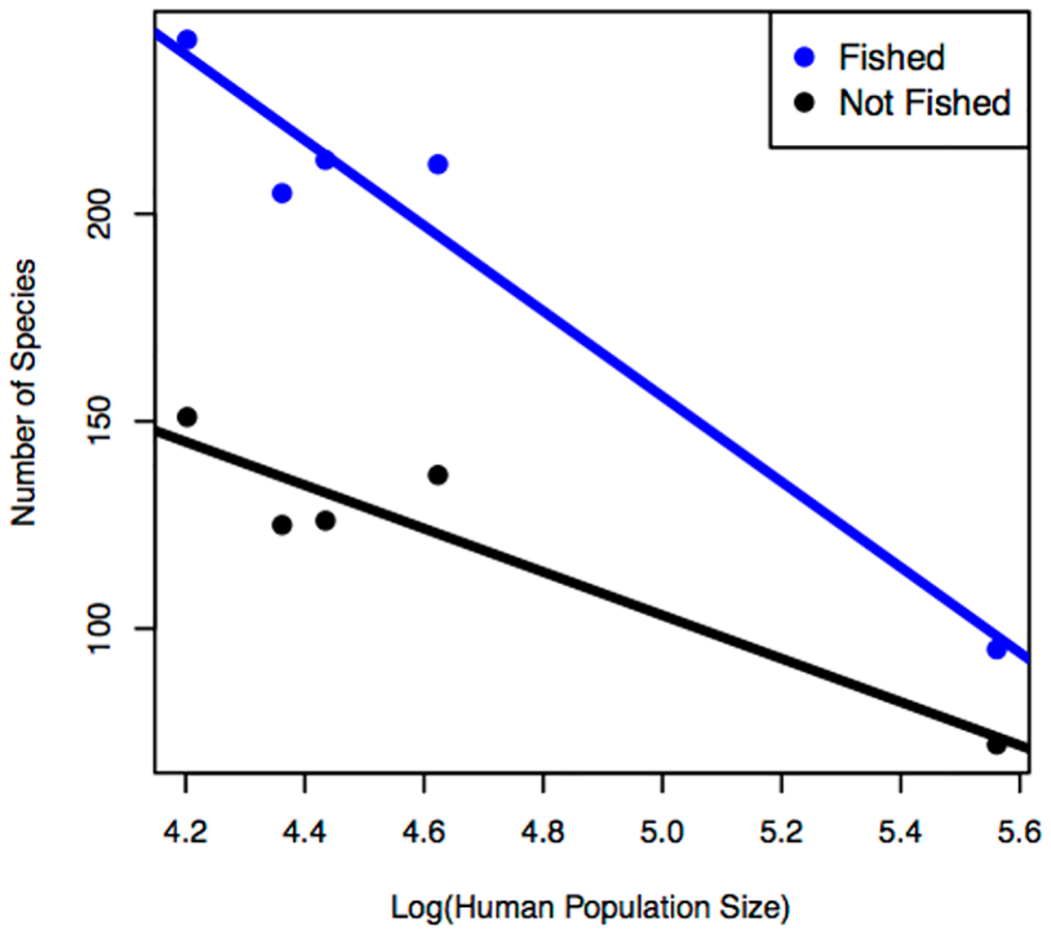
Regression analyses of human population size and the number of either fished or not-fished species. Human population has been log transformed.

Species richness decreased with human population size, and that decrease was significantly stronger for fished species (slope = -44.7, 95% CI of slope -51.6 to -37.8) than for non-fished species (slope = -23.1, 95% CI of slope -29.8 to -16.4) (Fig 2). Excluding Bootless Bay from the analysis revealed the same basic pattern of a more rapid decrease in the species richness of fished species (slope of -91.7) than in non-fished species (slope of -55.3), but as sites are replicates, the models with n = 4 sites lack statistical power and were not significant.

Total beta diversity (Jaccard distance) was highest between Bootless Bay and all other sites for both the non-fished and fished datasets (Table 2). Beta diversity was larger among sites for the fished dataset than the non-fished dataset via paired t-test (t = 11.87, P < 0.0001). When decomposed into richness and replacement components, the majority (73–91%) of the large beta diversity between Bootless and the other sites was due to the richness component. In contrast, the majority of the beta diversity among the sites other than Bootless Bay were in the replacement partition for most reef pairs. The replacement component of beta diversity (Breplace) was also larger for fished datasets than for non-fished dataset via paired t-test (t = 2.98, P = 0.015).

**Table 2:** Beta diversity between reef sites calculated using Jaccard dissimilarity (B_total_), with distances partitioned into beta diversity due to richness (B_rich_) and replacement (B_replace_). Diversity was calculated on “Fished”(>30 cm SL) and “Non-Fished” (<30 cm SL) species separately.

	Fished					Non- Fished				
		Bootless	Bismark	Kimbe	Madang		Bootless	Bismark	Kimbe	Madang
B_total_	Bismark	0.624				Bismark	0.53			
	Kimbe	0.631	0.341			Kimbe	0.544	0.232		
	Madang	0.659	0.358	0.345		Madang	0.539	0.242	0.211	
	Milne	0.653	0.297	0.286	0.327	Milne	0.561	0.253	0.213	0.233
B_replace_	Bismark	0.119				Bismark	0.134			
	Kimbe	0.107	0.309			Kimbe	0.147	0.225		
	Madang	0.148	0.331	0.342		Madang	0.084	0.161	0.136	
	Milne	0.064	0.152	0.173	0.213	Milne	0.052	0.089	0.052	0.147
B_rich_	Bismark	0.505				Bismark	0.396			
	Kimbe	0.524	0.031			Kimbe	0.397	0.007		
	Madang	0.511	0.028	0.004		Madang	0.455	0.081	0.075	
	Milne	0.59	0.144	0.113	0.114	Milne	0.51	0.165	0.161	0.086

The calculated NODF metrics for nestedness of fish communities at all five sites were all significantly lower than simulated metrics for all analyses, indicating that these datasets are significantly anti-nested (Table 3). Both columns (sites) and rows (species) contributed to these patterns. When Bootless was removed from the analyses, the anti-nested pattern disappeared for non-fished species, and there was no significant pattern for the NODFc (representing the contribution of sites to the nestedness patterns) for the other two datasets.

**Table 3:** Results of nestedness analyses. NODF stands for “Nested metric based on overlap and decreasing fill” for the whole data set, NODFc focuses on individual sites, and NODFr focuses on species.

	All sites			Sites excluding Bootless Bay		
	Calculated metric	Z score	*P*	Calculated metric	Z score	*P*
**All Species**						
NODF	70.17	-3.89	<0.0001	58.94	-1.83	0.034
NODFc	87.64	-3.68	0.00011	87.14	0.19	NS
NODFr	70.16	-3.95	<0.0001	58.94	-0.95	NS
**Fished**						
NODF	69.28	-2.94	0.0016	60.43	-1.67	0.047
NODFc	85.29	-2.78	0.0026	84.25	-0.04	NS
NODFr	69.28	-3.23	0.0006	60.43	-5.61	<0.0001
**Not Fished**						
NODF	70.67	-3.34	0.0004	54.90	0.49	NS
NODFc	91.38	-2.46	0.0068	92.02	0.34	NS
NODFr	70.66	-2.94	0.0016	54.88	0.53	NS

There were 76 species in the museum holdings collected by Andrew Goldie between 1881 and 1891, including seven species that were not found on the list of species found in the modern data (Table 4). Goldie collected these using a combination of nets, hand lines, and purchases from villagers [25]. Of the 69 species that are shared between the historical and total modern data lists (from all five sites), 33 are not present in Bootless Bay. The ratio of fished: non-fished species in the extirpated species, the 33 fishes present in 19^th^ but not the 21^st^ century Bootless Bay, was 3.7, which is significantly higher than the ratio of fished:non-fished species across the entire historical Bootless Bay data set (χ^2^ = 6.08, *P* = 0.01). This indicates that fished species were proportionally more abundant in the past. The ratio of fished:non-fished species in the 36 extant species (fishes present in both 19^th^ and the 21^st^ century Bootless Bay) was 0.7, and not significantly different from the expected ratio based on the historic data set as a whole (Χ^2^ = 2.81, *P* = 0.09).

**Table 4:** Species found in museum collections from 1881–1889 not present in modern species datasets at any of five sampled reef sites. Maximum Length is reported for the Standard Length (tip of snout to the posterior end of the midlateral portion of the hypural plate).

Genus	Species	Family	Maximum Length (cm)
*Alectis*	*ciliaris*	Carangidae	150
*Atule*	*mate*	Carangidae	30
*Trachinotus*	*blochii*	Carangidae	110
*Lutjanus*	*monostigma*	Lutjanidae	60
*Cephalopholis*	*miniatus*	Serranidae	50
*Epinephelus*	*longispinus*	Serranidae	55
*Siganus*	*canaliculatus*	Siganidae	30

Sharks and groupers, which are highly sensitive to fisheries pressure due to their large size and slow reproductive rate, showed a negative relationship with human population size (Table 5). Bootless Bay had only the two smallest of nine total shark species (22%) and only 14 of 48 epinepheline serranid species (29%). Madang, the second largest city in our analysis (2011 population ~42,000), had 34 of 48 (70%) epinepheline serranids but only two of nine sharks (22%).

**Table 5:** Number of species (and percent of total number of species in that group) for indicator taxa found at each of five reef sites in Papua New Guinea. Groupers include all epinepheline serrandis, sharks include members of both Carcharhinidae and Sphyrnidae.

	Bootless	Bismark Sea	Kimbe	Madang	Milne
Sharks	2	5	7	2	5
Groupers	14	33	37	34	40
% of all Sharks	22.2	55.6	77.8	22.2	55.6
% of all Groupers	29.2	68.8	77.1	70.8	83.3

## Discussion

Distinguishing among the different ways that humans impact species diversity is key to designing effective management aimed at preserving that diversity. By evaluating fished species separately from those not fished, we find that that both direct and indirect impacts of human populations are important in structuring beta diversity in this reef system. The correlation between decreasing species richness with increasing human population is consistent across datasets, but strongest for fished species, likely due to an increase in fishing pressure with human population size. We see that across all sampled sites, fished species tend to be less diverse than non-fished species (Table 1). This is particularly true for our highly sensitive taxa, sharks and groupers, where even at relatively low population sizes there was a strong relationship between human population size and the reduction of large bodied, fisheries targets (Table 5) [10]. Both taxa are important in commercial and artisanal fisheries [26,27] are subject to strong fisheries pressure from international trade [28,29] and appeared in fish markets throughout Papua New Guinea (Drew pers. obsv.). Our results show that even low human population sizes are sufficient to differentially impact community structure through the removal of large bodied (mostly predatory) species. Our work supports those of other authors in the region who have found that low intensity artisanal fisheries, preferentially remove large bodied fishes first and can drive down the average length of catch [10].

Indirect impacts of human development appear to play a significant role in determining species richness. Richness of non-fished species decreased with human population size, suggesting that additional factors are also driving community composition changes. Various stressors have be linked to reductions in diversity, including sedimentation [30], pollution [31], or nutrient loading [32]. While we do not have data to identify which of the indirect stressors (sedimentation, pollution, nutrient load etc.) are most important in in this community it is apparent that they are impacting entire reef ecosystems and warrant further study [33].

These stressors, both direct and indirect, have not only impacted the number of species present but also beta diversity. Fished datasets are significantly more distinct from each other than non-fished datasets. This pattern is partially due to the larger reduction in fished species richness from low human population to high human population sites. However, partitioning beta diversity into richness and replacement components demonstrated that beta diversity is still larger for the fished species after richness is partitioned. Larger fish tend to function at higher trophic levels and thus fisheries tend to take out predatory species preferentially [34]. Because there are fewer kinds of species at higher levels, any extirpation of a single species at a site will likely result in a disproportionately large decrease in beta diversity. Top level predators may be extirpated through random fishing pressure, or areas may have different cultural practices resulting in preferential removal of specific species [35,36].

Although both direct and indirect stressors structure these reef communities, their relative importance varied along the population gradient. The difference in richness of fished species and non-fished species was greater at the sites with smaller human populations; fishing was a strong factor in reducing diversity, even when those fisheries were small scale, localized, and artisanal [10]. As human populations grow, the relative importance of habitat degradation, via indirect stressors, grows [37]. Thus, for areas with large human populations, management strategies need to focus just as much on their general environmental quality as well as on fishing pressure, while in areas with smaller populations, the establishment of a system of interconnected no-take reserves will provide an appropriately tailored conservation measure and is likely to be most effective.

Among our datasets, the fish community in Bootless Bay was distinct, driving many of the statistical relationships we found. Species richness at Bootless Bay was half that of the next most depauperate site (Bismark Sea). Bootless Bay reefs are likely to have a lower species diversity due to a combination of fisheries and non-fisheries stressors, brought about by their location downstream of the city of Port Moresby, population ~365,000 in 2011. Port Moresby is the largest city in Melanesia. Several forms of perturbation have occurred as a result of such a large human population, including high levels of siltation [32], loss of mangrove cover due to conversion for building material [33], and high levels of marine debris on its beaches (upwards of 17.5 kg/m) from domestic sources [33].

The current low species richness of Bootless Bay can be placed in a historical context when compared with data from 19^th^ century museum collections. In 1880, Port Moresby in Bootless Bay was an isolated colonial outpost with an approximate population of 2,000 people and only localized fishing pressure [38]. Historical collections show that, prior to the stressors that arose from large human population size, Bootless Bay once maintained a diverse species pool including large fish that are absent today. These data also demonstrate the extirpation of fish from the region. There were seven species of fishes recorded in the historical data set that are not present in any of the modern datasets. These species have an average maximum length of 69.2 cm suggesting that they would all have been fisheries targets (Table 4). Most of these species are coral or brackish water associated species and at least one of these species, *Siganus canaliculatus*, is an important consumer of macroalgae. Therefore, these extirpations could have contributed to environmental degradation of Bootless Bay [39]. Of the seven species, six have ranges that include Papua New Guinea (although, perhaps tellingly, none of those were found in any of our sample sites), while one, *Epinephelus longispinis* (Kner 1864), is now only currently distributed as far east as the Banda Sea [40], approximately 2,200km away from Bootless Bay.

Although modern and historical data indicate that Bootless Bay is a highly stressed and relatively depauperate fish community, it is still valuable for regional conservation. Our data indicate that Bootless Bay is not simply supporting only the most common fish species across these reefs; the Bootless Bay community drives the pattern of significant anti-nestedness we found. In fact, twelve species are either found only at Bootless Bay or in only one other site. This has important implications for regional conservation, as it reveals a potential trade-off; if efforts concentrate on protecting the more diverse sites, the unique assemblage near Bootless Bay may be lost [41].

Whenever data collected by multiple individuals across a large, culturally diverse region are combined for analysis caution is warranted. The reefs sampled here cover eight degrees of latitude, multiple microhabitats, and variable current regimes, which could all affect species richness and reef community composition. Additionally, while we selected data to minimize the number of collectors, it is impossible to avoid some degree of variability in the sampling techniques across our sites. Consistent sampling using equivalent methodologies should be performed across these sites to confirm the patterns we report here, and to extend our analyses to cryptic and hard-to-identify species.

Just as the data and sites differ, so do the associated human populations. This is relevant when considering how to translate our findings into management. Papua New Guinea is one of the most culturally diverse regions in the world. Not all individuals have access to marine resources, nor do all individuals target the same marine resources [37]. Furthermore, low-density human populations could still have substantial impacts on marine ecosystems via intensive agriculture [42] Because of this heterogeneity, we used human population as an indicator of human impacts on fish communities, but clearly further research into the ethnographic and political ecology of the region will be necessary to enact the most effective conservation programs [39]

Here we show the multifaceted impacts that human population pressure can have on targeted and non-targeted species. Our conclusions are that 1) anthropogenic stressors impact both targeted and non-targeted species; 2) high human population pressure will drive a decrease in fished species diversity faster than non-fished species; and 3) management of biodiversity near high human population sites may have to focus more on environmental stress than sites with low human population pressure. While our focus was the reefs of Papua New Guinea, these results should prove useful to ecologists and conservation biologists working across a variety of systems.

In this paper we highlight not only the ability to detect fisheries pressure from presence / absence lists but we also show the importance of developing, curating, and publishing high quality collections-based information. Species distributional data underpin much of modern ecological analyses; such data are the raw material that allow us to understand the ways in which communities assemble and interact. Natural History museums, as repositories of collections based information, play a critical role in our ability to understand the temporal and spatial extant of variation in biodiversity, and to quantify the anthropogenic influences on that biodiversity. As museums continue to digitize their existing collections the utility of these biodiversity repositories will increase, ultimately providing enhanced temporal and spatial coverage for conservation managers and ecologists seeking to understand the vivid splendor of biodiversity [43].

## Supporting Information

S1 Table A list of all species used in analyses. (CSV)
